# Apoptotic mechanism of propofol-induced developmental toxicity in zebrafish embryos

**DOI:** 10.1371/journal.pone.0286391

**Published:** 2023-05-30

**Authors:** Yali Ge, Wenjuan Yuan, Wenzhu Jia, Zhongxia Guan, Tianfeng Huang, Yang Zhang, Chengyi Song, Yinggang Xiao, Ju Gao

**Affiliations:** 1 Department of Anesthesiology, The Second Xiangya Hospital, Central South University, Changsha, Hunan, China; 2 Department of Anesthesiology, Yangzhou University Affiliated Northern Jiangsu People’s Hospital, Yangzhou, Jiangsu, China; 3 Yangzhou Key Laboratory of Anesthesiology, Yangzhou, Jiangsu, China; 4 College of Animal Science and Technology, Yangzhou University, Jiangsu, China; Jiangsu University, CHINA

## Abstract

General anesthetics can cause neurological damage and long-term behavioral/cognitive impairment during fetal and early postnatal life. However, the adverse influence on embryo development induced by propofol is unclear. We used embryonic zebrafish to explore the effects of propofol on embryonic and larval growth and development, and the related apoptotic mechanism. Zebrafish embryos were immersed in propofol (1, 2, 3, 4, and 5 μg/ml) dissolved in E3 medium from 6 to 48 hours post fertilization (hpf). The survival rate, locomotion, heart rate, hatchability, deformity rate, and body length were analyzed at defined stages. Terminal deoxynucleotidyl transferase nick-end-labeling was used to detect zebrafish embryo apoptosis, and the expression levels of apoptosis-related genes were determined using quantitative real-time reverse transcription PCR and whole-mount *in situ* hybridization. Larvae at 48 hpf were anesthetized by immersion in E3 culture medium containing 2 μg/ml propofol, the reasonable anesthetic concentration for zebrafish embryos, which caused significant caudal fin dysplasia, light pigmentation, edema, hemorrhage, and spinal deformity, and decreased the hatchability, body length, and heart rate. The numbers of apoptotic cells in propofol-treated 12, 48 and 72 hpf embryos increased significantly, and the mRNA expression levels of intrinsic apoptosis pathway-related *casp3a*, *casp3b*, *casp9*, and *baxb* genes were upregulated, mainly in the head and tail. Propofol decreased apoptosis in the head and back of 24 hpf zebrafish, which was consistent with the mRNA expression analysis. Our findings demonstrated that zebrafish embryos and larvae exposed to propofol experienced developmental toxicity, which correlated with the intrinsic apoptosis pathway with *casp3a*, *casp3b*, *casp9*, and *baxb* as the key genes.

## Introduction

In the United States, more than 3 million infants and children accept general anesthesia in surgery every year, and the incidence of non-obstetric surgery during pregnancy in Europe and the United States is about 0.75% to 2% [1]. Internationally, whether general anesthesia adversely affects the growth and development of the fetus has been a hot issue for anesthesiologists. The Food and Drug Administration warned that exposure of pregnant women to general anesthetics, such as propofol, might harm the brain development of their fetus in the third trimester of pregnancy [2]. To date, there have been few clinical studies on the effects of propofol on early fetuses. One case reported that the fetus of a woman at 14 weeks gestation was deemed nonviable after the mother’s administration of propofol for sedation [3]. The clinical consensus is to avoid general anesthesia in early pregnancy if possible, as the effects of even the latest anesthetics, such as propofol, on fetal organogenesis are not fully understood [4,5]. However, *in vivo* animal experiments have shown that fetuses experience neurological damage and long-term behavioral cognitive impairment after general anesthesia using propofol [6–9].

Apoptosis, the first discovered type of programmed cell death (PCD), is an important means of removing excess cells during the early development of the fetus and plays an important role in the normal development of organisms [10,11]. Experiments, including those in non-human primates, have shown that general anesthesia maintained by inhalation anesthetics aggravates apoptosis during neurodevelopment, leading to acute brain injury [12–14]. For propofol, as a commonly used intravenous general anesthetic in non-obstetric and fetal surgery during pregnancy, there is a lack of strong clinical evidence demonstrating its neurotoxicity; however, its effects on neuronal apoptosis in mammalian fetuses have been frequently reported [7,8,15,16]. When anesthetizing mid-gestational rats with propofol, Chen et al. [7] found that the offspring developed neuronal damage both *in utero* and after birth, exhibiting depression- and anxiety-like behaviors and cognitive impairment. Kinesin family member 17 (KIF17) is a neuron-localized directional motor protein involved in dendrite-directed material transport [17]. A study found that propofol exposure in rats at 14 days of gestation resulted in learning and memory deficits in the offspring that might be related to KIF17 [16]. In addition, an experiment on fetal rhesus monkeys, which are highly similar to humans, found that pregnant monkeys gave birth to offspring with significantly increased neuronal apoptosis after propofol anesthesia [8]. However, the molecular mechanisms active during the lengthy use of propofol on brain development are poorly understood. Research on the influence of propofol on apoptosis in the early developing nervous system has not been carried out effectively. This is because of the limitations concerning the placental barrier between fetal rats and their mothers, as well as the difficulty in observing the developmental process of fetal rats directly. In addition, the adverse effects on embryo development induced by propofol have received less research attention.

An ideal model system to investigate developmental toxicology is provided by the zebrafish embryo, because their external post-fertilization, quick development, transparency of embryonic stage, and rapid maturation make studying their embryogenetic mechanism easier than studying mammalian fetuses [18]. In recent years, zebrafish embryos and larvae have emerged as a good model to study the mechanism of general anesthesia of propofol and clinical phenomena such as delay of anesthesia because they have the same drug-metabolizing enzymes as humans [19–21] and their behavior, such as color preference, swimming rate, and anchorage tropism (the tendency to stay close to borders of the petri-dish) [22–24]. Propofol exposure would lead to delayed embryo incubation, cardiac and retinal developmental toxicity, and zebrafish learning and memory ability impairment [25–28]. Related studies revealed that inhibition of the electron transport chain increased neuron apoptosis and the delayed myelination induced by reduction of myelin basic protein (MBP) played an important role in this toxic process [27,28].

The gene homology between zebrafish and human is as high as 87%, and the apoptosis-related genes are highly conserved [29,30]. Terminal deoxynulceotidyl transferase nick-end-labeling (TUNEL) staining and whole-mount *in situ* hybridization (WISH) can be used to observe the distribution of apoptotic cells and the trend of related genes, which effectively makes up for the deficiency of mammalian models [31,32]. As the most important programmed cell death in development, apoptosis affected by propofol during the development of zebrafish was only briefly analyzed by acridine orange (OA) and TUNEL staining in several articles [27,28], thus the molecular mechanism require further exploration.

Above all, previous studies primarily evaluate the effects of propofol on neuronal apoptosis in mammalian fetus. Therefore, our goal was to comprehensively observe the effects of propofol exposure on the growth and development of embryonic zebrafish, and identify key apoptosis genes using TUNEL staining, quantitative real-time reverse transcription PCR (qRT-PCR) with two reference genes, and WISH techniques. Our was to provide potential molecular targets for the clinical prevention of anesthetic toxicity on fetal and neonatal development, and enhance the clinical safety of propofol.

## Methods and materials

### Breeding of zebrafish

The wild-type zebrafish Tuebingen (TU) strain, aged 3–4 months, was purchased from China Zebrafish Resource Center (CZRC, Wuhan, China). In a controlled environment with water at 28.5°C (14h day/10h night), separated by sex, zebrafish were fed with fresh brine shrimp at 8:00 am and 8:00 pm. Once a week, after 8:00 pm, male and female zebrafish (1:1) were placed in breeding boxes, which were separated by a transparent baffle. The following day, the light was turned on at 8:00 am, the water in the breeding boxes was changed, and the baffle was removed. After 15 minutes, zebrafish embryos were collected in petri dishes with E3 culture medium and placed at 28.5°C in biochemical incubator (PYX-280S-C, Keli Instrument, Ningbo, China). All treatments and protocols in this study were carried out strictly in accordance with the guidelines of CZRC and the Animal Experiment Ethics Committee of Yangzhou University (No. YZUDWSY201611-201).

### Intervention with the embryos

At 6 hours post fertilization (hpf), dead embryos (identified by the formation of white flocculent clumps or no heartbeat) were removed from the petri dishes and others were transferred to one petri dish for mixing, which reduced the impact of different F1 generations. Under a stereoscopic microscope (SZX7, Olympus, Tokyo, Japan), 6 hpf embryos at the gastrula period of development, which forms the germ ring and embryonic shield, resembling a semi-ring structure, were transplanted into 90 mm petri dishes [33]. With 50 embryos and 40 ml culture medium per dish, embryos were randomly divided into control and propofol groups.

A total of 2548 embryos were utilized in this study. In the optimal concentration gradient experiment for propofol, six groups (Control, 1, 2, 3, 4, and 5 μg/ml propofol) were assessed, with three replicates. To assess the survival, injury, and deformity rates, three independent experiments were conducted with 50 embryos in each group. Hatchability observations were performed with four replicates. In this process, the heart rate and body length were recorded for seven embryos in each group, while the twisting number and movement after needling were recorded for 28 embryos in each group. Additionally, qRT-PCR experiments were conducted at 12, 24, 48, and 72 hpf with three independent experiments with 50 embryos in each group. WISH was performed at 48 hpf with 12 embryos in each group, using three replicates for each gene. The TUNEL assay was conducted at 12, 24, 48, and 72 hpf with 12 embryos in each group, also using three replicates.

He et al. has reported that exposure of 6 hpf zebrafish embryos to propofol until 48 hpf induced significant cell apoptosis in the brain in a dose-dependent manner [28]. Meanwhile, two articles about propofol induced neurotoxicity in zebrafish used the same timeframe [27,34]. The reported EC50 for inhibition of the photomotor response was about 10 μM (1.78 μg/mL) in zebrafish larvae [35].There is about 1–3 μg/ml propofol in human blood during general anesthesia and entry into the anesthetized state usually takes a few minutes [36], thus, a 1–5μg/ml range was chosen for propofol during our primary concentration gradient experiment. In the propofol group, pure propofol solution (Macklin, Shanghai, China) was dissolved in E3 medium containing 0.014% dimethyl sulfoxide (DMSO; Sigma-Aldrich, St. Louis, MO, USA), and the final concentrations of propofol were 1, 2, 3, 4, and 5 μg/ml, respectively. The control group received only E3 medium. At 24 hpf, the medium for the propofol groups and control group were replaced with 20 ml of E3 medium, with or without propofol. Treatment with propofol was stopped at 48 hpf by replacing the culture medium with E3 medium without propofol. At 72 hpf and 96 hpf, 20 ml of fresh E3 culture medium of all groups was renewed, and dead larva were removed at each change of medium.

### Developmental toxicity assay

Developmental toxicity in zebrafish embryos and larva were observed as spinal curvature, hemorrhage, pericardial edema, light pigmentation, and yolk sac edema [31,37]. At each critical time point, the number of viable embryos, damaged embryos, and hatched embryos per dish were divided by 50, and converted into percentages to obtain the survival rate, the damage rate, and the hatching rate. In addition, the writhing times of 24 hpf embryos were observed under the microscope, and the reaction of 48 hpf embryos were observed when the tail was pricked. A camera phone (Vivo Z5x, Shenzhen, China) captured images of the embryos from the microscopic ocular, and after slow playback, the auxiliary counter counted the number of heartbeats in one minute, to provide the heart rate. Tricaine was added to the petri dish [38], and without response to touch in the tail with a needle, the larva body length was adjusted under a microscope to the lateral position so that their eyes overlapped. Zebrafish in propofol group did not need tricaine, but all embryos without hatching needed manual stripping (discarding the chorion from the embryos using ophthalmic forceps under a stereo microscope).

### *In situ* TUNEL assay

From 24 hpf, zebrafish were housed in E3 medium with 0.003% PTU (1-Phenyl-2-thiourea, Sigma-Aldrich) to prevent melanin deposition. Peeled using ophthalmic forceps if necessary, embryos were fixed in 4% paraformaldehyde (Solarbio, Beijing, China) at 4°C overnight, followed by gradient dehydration in methanol, and stored at −20°C for at least 2 hours. Zebrafish were rehydrated with serial dilutions of methanol. The apoptotic cells were then examined using a One Step TUNEL Apoptosis Kit (Elabscience, Wuhan, China); the nuclei were counterstained using 4′,6-diamidino-2-phenylindole (DAPI). Images were captured using a stereoscopic fluorescence microscope (M165FC, Leica, Wetzlar, Germany), and analyzed using Image J (NIH, Bethesda, MD, USA). Moreover, nonspecific uptake of fluorescent substances or autofluorescence in the yolk, iridophore, hatching gland, and neuromast represented false positive TUNEL staining and were excluded [39].

### RNA extraction and qRT-PCR

Zebrafish at 12–72 hpf from the control and propofol groups were collected, RNA was extracted using an RNA Easy Fast Tissue Kit (TIANGEN, Beijing, China), and then reverse-transcribed to cDNA in accordance with instructions of the FastQuant RT Kit (With gDNase) (TIANGEN). Each qPCR reaction was detected in a 7500 Real-Time PCR System (Applied Biosystems, Foster City, CA, USA). The cDNA sequences of 18 related genes were obtained through NCBI (https://www.ncbi.nlm.nih.gov/), and primers were designed using Primer3 (https://bioinfo.ut.ee/primer3-0.4.0/) (S1 Table). For each gene, *actb1* and *ef1a* were used as internal reference genes [40], and the normalized mean expression level was displayed (mean expression was divided by mean expression of 12 hpf zebrafish in the control group).

### Whole-mount *in situ* hybridization

RNA probes for *casp3a*, *casp3b casp9*, and *baxb* were generated from the zebrafish cDNA sequences using primer sequences, in which the T7 polymerase promoter was added to the 5′ end of the reverse primers (S2 Table). The digoxigenin (DIG)-labeled RNA probes were synthesized by PCR amplification and transcribed using T7 polymerase (Beyotime, Shanghai, China). WISH was performed as described previously [41]. Briefly, embryos were treated with 0.003% PTU to prevent pigmentation at 24 hpf and then collected at 48 hpf. Embryos were fixed in 4% paraformaldehyde (Solarbio) at 4°C overnight. Permeabilization and hybridization of the embryos with 80 ng of DIG-labeled RNA probes (Roche, Mannheim, Germany) was carried out overnight at 70°C, followed by incubation with anti-DIG antibody alkaline phosphatase, staining with nitro blue tetrazolium (NBT)/5-Bromo-4-chloro-3-indolyl phosphate (BCIP) (Roche, Indianapolis, IN, USA), and image capture using a stereoscopic microscope (M165FC).

### Statistical analysis

Data that basically satisfied the normal distribution and satisfied the homogeneity test are presented as the mean ± SD and were processed using an independent sample t-test, two-way analysis of variance (ANOVA) or repeated measures ANOVA, according to the data type. By contrast, after the data transformation attempt failed, data in the form of medians with lower and upper quartiles were subjected to two independent samples nonparametric tests and multiple independent samples nonparametric tests. Post-hoc multiple comparisons between groups were performed using Bonferroni tests. All statistical analyses were performed using SPSS 23 (IBM Corp., Armonk, NY, USA). Significance was set at P < 0.05 (two-tailed).

## Results

### Anesthetic Effect of propofol in zebrafish embryos

**Fig 1:**
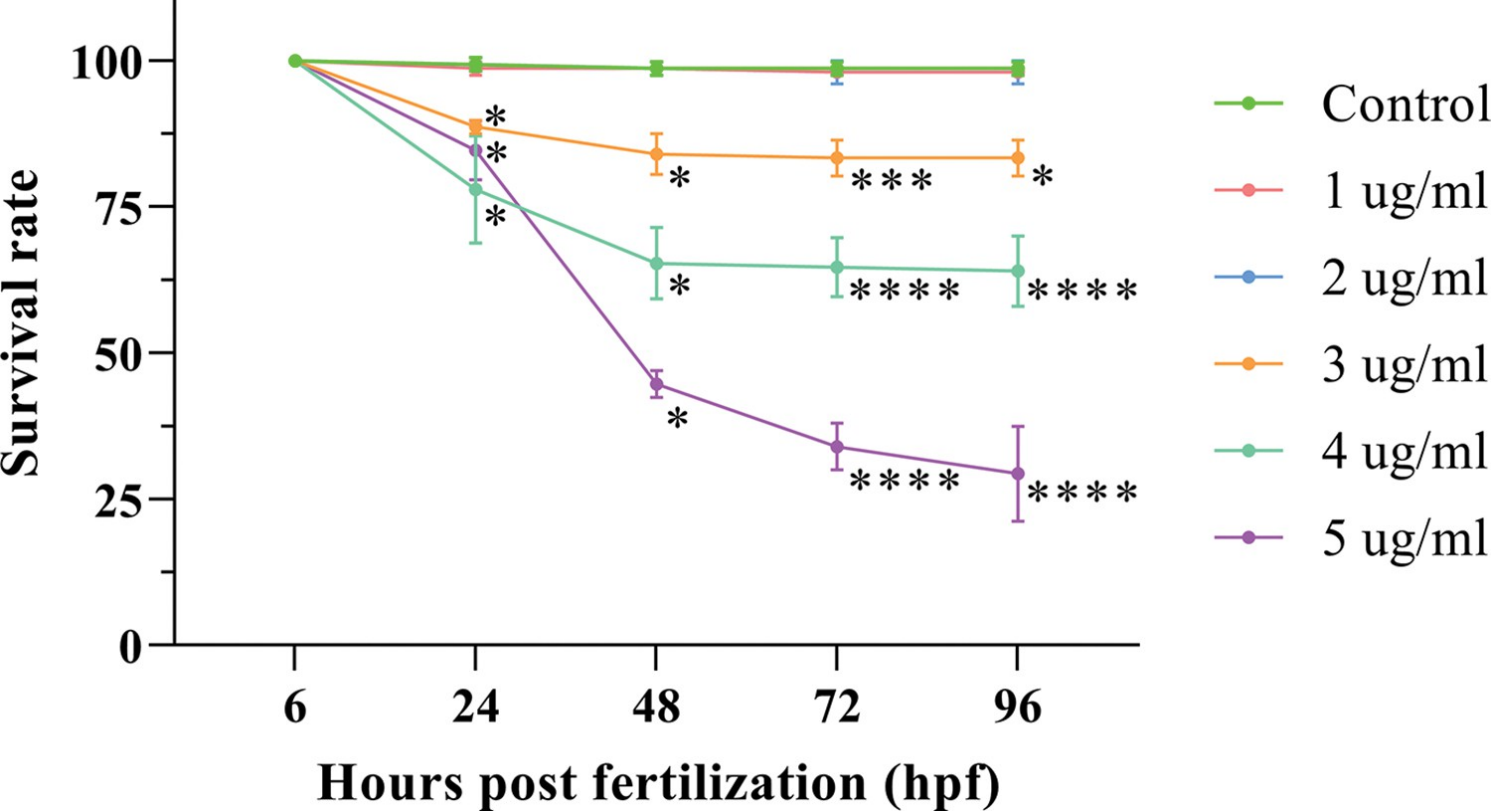
Effects of different concentrations of propofol on the survival rate of zebrafish embryos and larvae. Statistical analyses were performed between different time points, and data are shown as the mean ± SD. At 24 and 48 hours post fertilization (hpf), the data were compared by multiple independent samples nonparametric tests with Bonferroni correction. At 72 and 96 hpf, ANOVA with Bonferroni correction was used. * indicates that the related group was compared with control group at the same time point. Three independent experiments of 50 embryos for each group were carried out. Three independent experiments were carried out. *P < 0.05, **P < 0.01, ***P < 0.001, ****P < 0.0001.

Compared with the control group, in which less than two larvae died, the survival rate of embryos in the 1 μg/ml and 2 μg/ml propofol groups did not change significantly within 96 hpf (Fig 1). However, the survival rate of zebrafish in the 3, 4 and 5 μg/ml propofol groups showed a concentration-dependent decrease from 24 hpf. At 24 hpf, the embryos in the control group twisted 2–5 times per minute; however, no such behavior appeared in all the propofol groups at this stage. The embryos in the 2, 3, 4, and 5 μg/ml propofol groups did not respond to touch with a needle, while the embryos in the 1 μg/ml propofol and control groups showed body movement at 48 hpf (Table 1). The no observed effect concentration was less than 1 μg/ml, because the embryos did not twist compared with those in the control group. Having a complete anesthesia effect on zebrafish and little effect on their survival rate, 2 μg/ml propofol was chosen as the intervention to perform subsequent experiments.

**Table 1:** Effects of anesthesia on zebrafish locomotion (n = 28).

		Movement after needling		
Group	24 hpf (twisting number)	48 hpf	72 hpf	96 hpf
Control	5 (4, 5)^a^	Yes^b^	Yes	Yes
1 μg/ml	0 (0, 0)	Yes	Yes	Yes
2 μg/ml	0 (0, 0)	No^c^	Yes	Yes
3 μg/ml	0 (0, 0)	No	Yes	Yes
4 μg/ml	0 (0, 0)	No	Yes	Yes
5 μg/ml	0 (0, 0)	No	Yes	Yes

Four independent experiments of seven embryos for each group were carried out.

^a^Data are described by the median with lower and upper quartiles.

^b^All the zebrafish moved.

^c^None of the zebrafish moved.

hpf, hours post fertilization.

### Propofol at 2 μg/ml caused a developmental disorder of zebrafish

**Fig 2:**
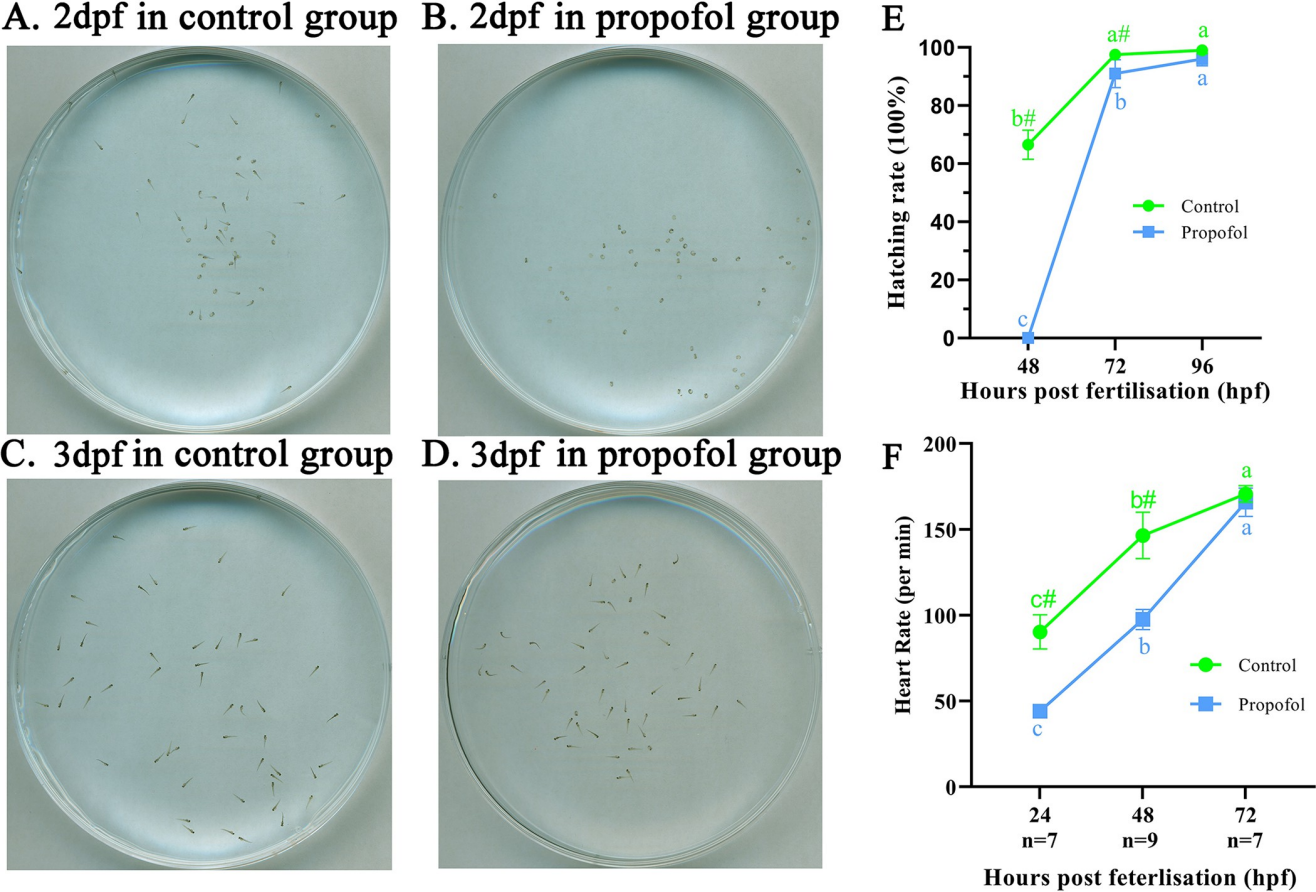
Hatchability and heart rate between two groups. (A-D) 48 and 72 hpf zebrafish in 90 mm dishes of the two groups. (E) The hatchability of zebrafish at each stage; Four independent experiments of 50 embryos for each group were carried out; ANOVA with Bonferroni correction. (F) Heart rate of zebrafish at each stage; n ≥ 7 for each group; two-way ANOVA with Bonferroni correction. Different letters indicate a significant difference between different time points for the same group (P < 0.05). # indicates a significant difference between the control group and the 2 μg/ml propofol group at this time point (P < 0.05). dpf, days post fertilization.

We next investigate whether the process of development was affected by 2 μg/ml propofol. Compared with that in the control group, the hatching rate in zebrafish exposed to propofol was markedly lower at 48 and 72 hpf (P < 0.05), while no significant difference was found between the two groups at 96 hpf (Fig 2A–2E). Moreover, 6–48 hpf zebrafish exposed to 2 μg/ml propofol showed a statistically lower heart rate than the control zebrafish; whereas, no statistical significance was displayed between two groups without propofol exposure at 72 hpf (Fig 2F).

**Fig 3:**
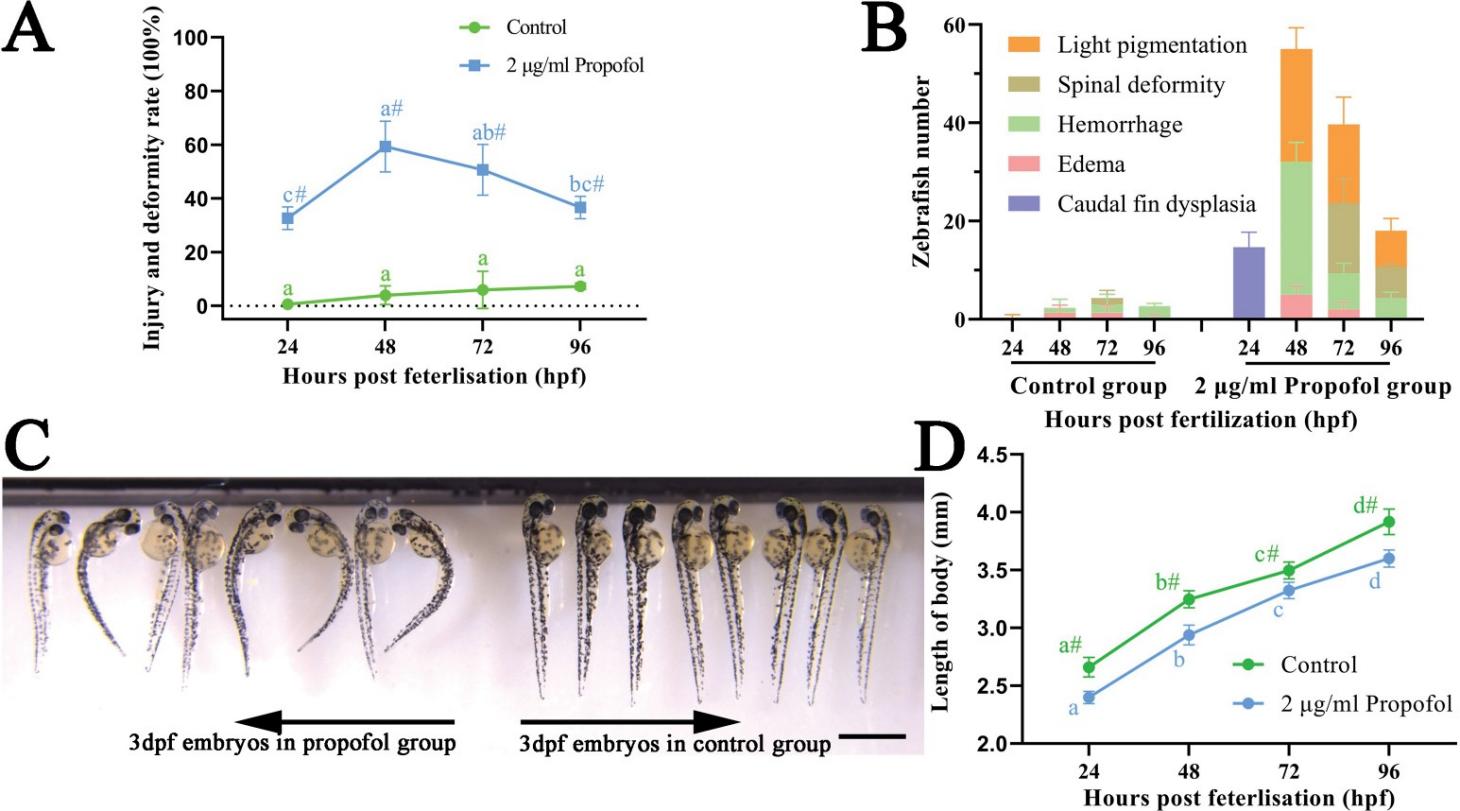
Developmental toxicity induced by 2 μg/ml propofol. (A) Injury and deformity rate from 24 hpf to 96 hpf in zebrafish. Three independent experiments of 50 embryos for each group were carried out; repeated measures ANOVA with Bonferroni correction. (B) Changes of specific types of deformities and injuries with the growth and development of zebrafish. Three independent experiments of 50 embryos for each group were carried out. (C) Image of 3 dpf zebrafish under a stereoscope microscope; scale bar = 1 mm; magnification = ×10. (D) Body length of zebrafish from 24 hpf to 96 hpf; n = 7 for each group; two-way ANOVA with Bonferroni correction. (A, D) No same letter indicates a significant difference between different time points for the same group (P < 0.05); # indicates a significant difference between the control group and the 2 μg/ml propofol group at this time point (P < 0.05).

From 24 to 96 hpf, at least 30% of the zebrafish exposed to 2 μg/ml propofol had obvious damage and deformity, including caudal fin dysplasia, hemorrhage, light pigmentation, and spinal deformity, among which the difference between the groups was the most remarkable at 48 hpf (Fig 3A and 3B). The developmental disorders and tissue damage were concentrated in the heart, yolk sac, or spine (S1 Fig). In addition, compared with that in the control group, the spinal deformity and light pigmentation of 3 days post fertilization (dpf) larvae in the propofol group were obvious (Fig 3C). However, larval body length was continuously and significantly inhibited during and after propofol exposure (Fig 3D). In brief, 2 μg/ml propofol might induce developmental disorders and tissue damage of zebrafish.

### Propofol influenced developmental apoptosis in zebrafish

**Fig 4:**
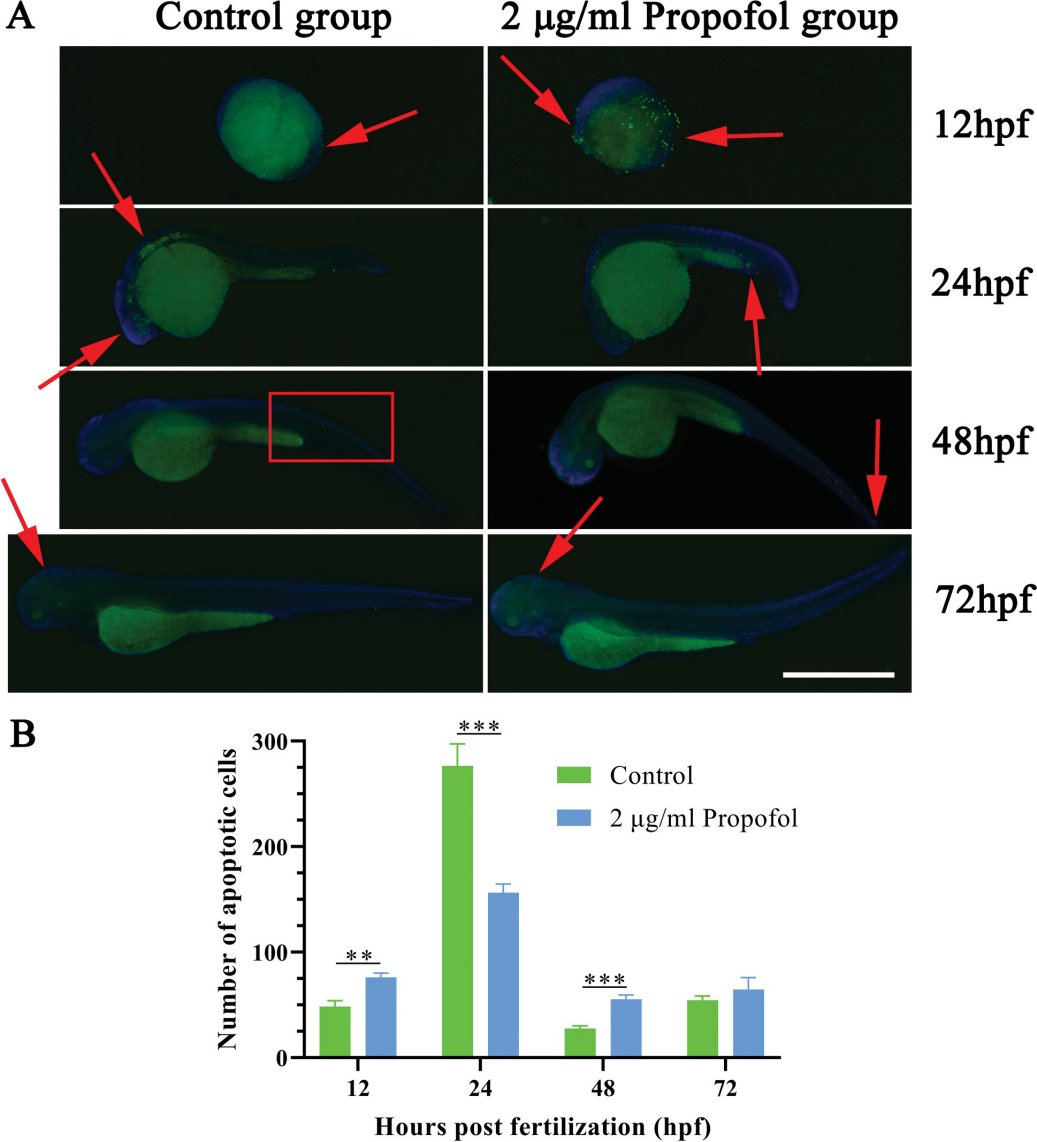
Result of terminal deoxynulceotidyl transferase nick-end-labeling (TUNEL) staining. (A) The merged image of signals for fluorescein isothiocyanate (FITC) (apoptotic cells) and 4′,6-diamidino-2-phenylindole (DAPI) (nucleus); red arrows and boxes represent site of apoptosis aggregation; scale bar = 1 mm, magnification = ×40. (B) Number of apoptotic cells; Independent sample t-test; n = 3; *P < 0.05, **P < 0.01, ***P < 0.001.

We then explored the potential correlation between apoptosis and the developmental disorders and tissue impairment induced by 2 μg/ml propofol. In the propofol group, more apoptotic cells gathered at the tail and some apoptotic cells showed at the head, while they were concentrated on the back and head in the control group (Figs 4A and S2). Compared with those in the control group, the 12 and 48 hpf zebrafish in the propofol group showed more apoptotic cells, while the trend was reversed at 24 hpf (Fig 4B). The results suggested that 2 μg/ml propofol disturbed the normal apoptosis process in zebrafish larvae.

### Expression dynamics of apoptotic genes

**Fig 5:**
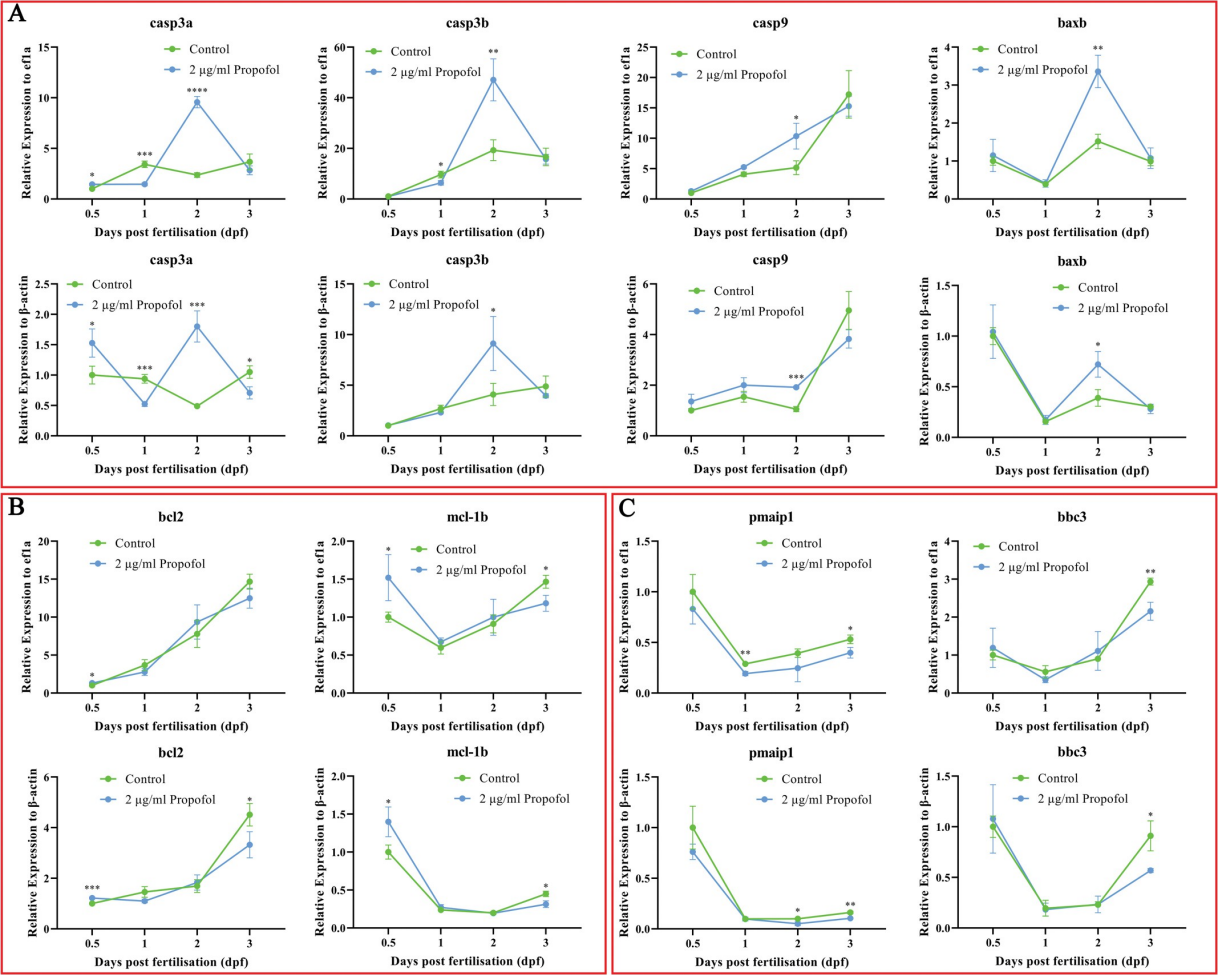
Apoptotic gene expression induced by propofol. (A) Pro-apoptotic gene expression significantly induced by propofol. (B) Anti-apoptotic gene expression induced by propofol. (C) The expression of pro-apoptotic genes only in the intrinsic pathway induced by propofol. An independent sample t-test was used to compare the mRNA expression in the two groups at the same time point. Three independent experiments were carried out. *P < 0.05, **P < 0.01, ***P < 0.001, ****P < 0.0001.

Considering apoptosis is a kind of complicated PCD, we further studied defined apoptotic genes to clarify the details of apoptosis affected by 2 μg/ml propofol. Overall, the mRNA expression levels of thirteen pro-apoptotic genes (*casp3a*, *casp3b*, *casp6*, *casp7*, *casp8*, *casp9*, *fadd*, *bida*, *tp53*, *pmaip1*, *bbc3*, *baxa*, and *baxb*) and five anti-apoptotic genes (*cflar*, *bcl2*, *bcl2l2*, *mcl-1a*, and *mcl-1b*) in the extrinsic or intrinsic pathway were evaluated using qRT-PCR in propofol-exposed and control embryos. Generally, after propofol exposure, significantly increased expression levels were observed for four key pro-apoptotic genes (*casp3a*, *casp3b*, *casp9*, and *baxb*) of the extrinsic and intrinsic apoptosis pathways, from 12 hpf to 3 dpf, but particularly at 2 dpf (Fig 5A). This coincided with the most obvious phenotypic variations (body length, hatching rate, and deformity) caused by propofol treatment (Figs 2 and 4). However, decreased expression levels were observed for *casp3a* and *casp3b* at 1 dpf after propofol exposure (Fig 5A). Downregulated expression levels were observed for the pro-apoptotic genes *pmaip1* and *bbc3*, which are the initial target molecules of the extrinsic apoptosis pathway, particularly at 3 dpf (Fig 5B). Downregulated expression levels were observed for anti-apoptotic genes *bcl2* and *mcl-1b* at 3 dpf; however, upregulated expression levels of *bcl2* and *mcl-1b* were observed at 12 hpf (Fig 5C). No significant differences in expression were observed for the other pro-apoptotic and anti-apoptotic genes (S3 Fig), while only the anti-apoptotic genes *bcl2* and *mcl-1b* were upregulated in propofol-treated 12 hpf embryos, and *mcl-1b* only was upregulated in 12 hpf and 3 dpf embryos. When the embryos were exposed to propofol up to 48 hpf, *casp3a*, *casp3b*, *casp9*, and *baxb* began to be upregulated in the embryos of the propofol group, while all the anti-apoptotic and some pro-apoptotic genes showed no significant difference.

### WISH analysis of key genes

**Fig 6:**
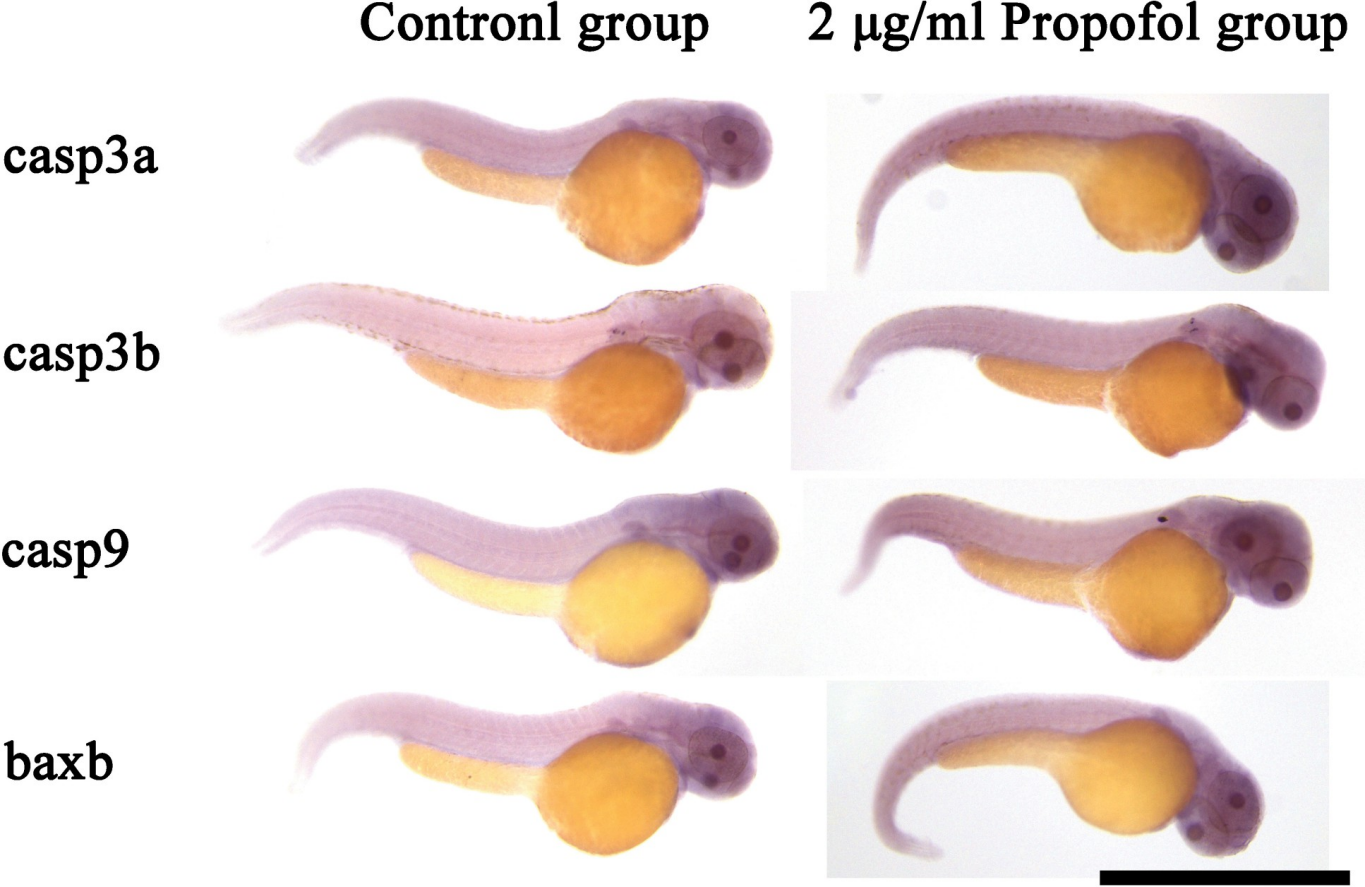
Result of whole-mount *in situ* hybridization at 48 hpf. The intensity of the color indicates the level of mRNA expression of the corresponding gene. Scale bar = 1 mm. Magnification = ×50.

To determine the actual activity of these key genes, the mRNA expression profiles of 48 hpf larvae, which expressed the most obvious difference, were investigated using WISH (Fig 6). All key genes of whole bodies in the propofol group were expressed at higher levels than those in the control group, in which *casp3a* and *casp3b* showed a significant difference that was consistent with the qRT-PCR results. In the head, heart, and digestive system regions, all key genes showed obvious mRNA expression, representing marked levels of apoptosis. In particular, more prominent mRNA expression of *casp9* and *baxb* was observed in the tail areas of 48 hpf zebrafish exposed to propofol, which explained the fluorescent spots in the TUNEL assay.

## Discussion

The results of this study suggested that propofol not only inhibited the movement of juvenile fish, but also hindered the development of embryos, which caused a hatching delay. The critical period of the development of various major organs in zebrafish is 6–48 hpf, which corresponds to the gastrula and segmentation periods [33]. In this period, the nervous system develops, the heart forms [33,42], and many biological pathways that are conserved between zebrafish and humans are activated [43]. Exposure to propofol in this stage caused zebrafish to develop significant toxic damage, indicating that zebrafish embryos can be used as a good model of propofol general anesthesia. In addition, the optimal concentration was 2 μg/ml propofol, which could produce a complete anesthetic effect, abrogated body movement, slowed the heart rate, and did not affect the zebrafish survival rate. During normal development, 2 dpf is the key time point for zebrafish embryos to hatch. Normal incubation of zebrafish requires the body to reach the appropriate stage of development, and it needs to twist itself to break the membrane [33]. The body length of zebrafish reflects their development [44,45]. During the development of zebrafish, melanin cells, yellow–orange xanthophores, and iridescent iridophores are produced, among which the melanin cell begins to deposit melanin at 24 hpf [46]. In addition, this embryonic/early larval pigment pattern is completed by 5 dpf and includes several stripes of black melanophores with yellow–orange xanthophores scattered widely over the flank [47]. In summary, the degree of pigmentation can reflect the growth status of zebrafish embryo and larvae. Clearly, our results demonstrated that 2 μg/ml propofol inhibited the development of embryos even after the end of propofol exposure.

Caudal fin observation indicated that propofol might only slow caudal fin growth rather than inhibit it completely. The lack of hemorrhage observed in the 24 hpf embryos might be because that zebrafish blood circulation occurs after 24 hpf [33]. The result revealed that zebrafish hemorrhage is more likely to be a manifestation of acute vascular injury caused by the accumulation of inflammatory factors, and the possibility of vascular dysplasia or malformation is less likely [48,49]. Many studies have shown that when zebrafish are stimulated by chemical substances or drugs in the environment, the spine is prone to curvature and deformity, which is an important indicator to observe pollutants and drug toxicity [27,37,50–52]. The 48 hpf zebrafish embryos in the propofol group did not hatch, making it difficult to observe spinal deformities; therefore, we could not rule out that there were a large number of zebrafish with spinal deformities. The results of the developmental toxicity experiment of propofol on zebrafish embryos showed that propofol caused acute damage to the embryo during continuous exposure; however, the acute injury would gradually heal itself after the end treatment. By contrast, the spinal deformity possibly persisted for a long time.

We found that propofol delayed development; however, after its removal, it seems that development continued and returned to normal values similar to the control. Rapid metabolism and suppression of the immune system are considered as potential reasons. Propofol is metabolized rapidly and entirely [53], which leads to movement, one of the key factors influencing hatchability. Moreover, the heart rate recovered to normal values quickly after the removal of propofol. In addition, investigators reported that propofol restrains immune system and cell activity in organisms [54–56]. When the inhibition was removed, the whole zebrafish accelerated the removal of damaged tissues and cells, eventually returning to normal development. Nevertheless, the discrepancy between the body lengths of the two groups showed no signs of diminishing, which might have resulted from sluggish bone growth progression.

PCD is a protective measure by which organisms respond to external dangerous stimuli or physiological stimuli [57]. Moderate PCD contributes to the development and homeostasis of multicellular organisms, while excessive PCD can cause tissue damage, enhance inflammation, and impair normal physiological functions and growth [58,59]. As an extremely important PCD, apoptosis plays an vital role in zebrafish early development by removing damaged, misplaced, or otherwise unwanted cells [29].

From 12 to 72 hpf, a certain amount of apoptosis played an important role in zebrafish embryonic development. At 12 and 24 hpf, excessive tail cell apoptosis induced by propofol might have affected the normal development of the caudal fin, leading to the failure of caudal fin emergence in some embryos. However, at 24 hpf, insufficient apoptotic cells in the head and back possibly led to the spinal deformity observed in 48 to 96 hpf embryos. In addition, the brain of 24 hpf zebrafish was formed and divided into the telencephalon and diencephalon, and the heart began to beat and the circulatory system was formed [33], which was possibly related to the high levels of apoptosis in the brain of 24 hpf control zebrafish. Moreover, the low density of apoptotic cells in the dorsum was probably related to the light pigmentation of the zebrafish. These results suggested that propofol altered the number and distribution of apoptotic cells during early development in zebrafish, and caused damage and deformity. After the withdrawal of propofol, although the effect on apoptosis disappeared, some injury and deformity remained. This might be explained as follows. Some studies showed that propofol caused developmental disorders in zebrafish through various mechanisms, such as altering the expression of genes related to heart development and function [26], inhibiting axon growth of motor neurons [34], and inhibiting the mitochondrial electron transport chain [28]. In addition, apoptotic death of some non-regenerative cells such as nerve cells, skeletal muscle cells, and cardiomyocytes potentially lead to persistent injury in juvenile zebrafish.

Apoptosis can be divided into the intrinsic apoptosis and death receptor-mediated extrinsic apoptosis pathways, which are initiated by different proteins and eventually lead to apoptosis through different pathways [60]. However, it can also release cytochrome C through mitochondrial membrane rupture mediated by the Bid protein, which triggers the mutual polymerization of Caspase-3, Caspase-6, and Caspase-7, causing plasma membrane blebbing, chromatin condensation, organelle disassembly, and formation of apoptotic bodies [61]. The qRT-PCR results for zebrafish embryos at different stages showed that propofol-induced apoptosis might be mainly related to pro-apoptotic genes, mainly in the intrinsic pathway. The results demonstrated that at the initial stage of propofol exposure, both pro-apoptotic and anti-apoptotic genes were activated and antagonized each other. Collectively, the results of qRT-PCR revealed that *casp3a*, *casp3b*, *casp9*, and *baxb* are the key genes in propofol-induced apoptosis. The conclusion was strengthened by the result of WISH, in which the brain and heart not only had the highest expression of key genes for apoptosis in the normal development of zebrafish, but also had the most obvious enhancement of gene expression in these organs after induction by propofol, which are the susceptible target organs for apoptosis.

The expression changes of pro-apoptotic genes, including *casp3a* and *casp3b*, at 12 to 72 hpf were consistent with the results of TUNEL staining, which demonstrated the effect of propofol on apoptosis during zebrafish development from the molecular perspective. More importantly, caspase activation is increasingly associated with physiological and anesthesia-induced pathological non-apoptotic outcomes that do not lead to neuronal death, but have major effects on cell morphology and function [62]. The expression of the caspase family at the transcriptional level increased with the development of zebrafish embryos; however, TUNEL staining showed that the number of apoptotic cells was highest at 24 hpf. One possible reason for this contrast is that the increased expression of caspase family molecules is not entirely related to the apoptotic pathway.

One limitation of the research is that no targeted inhibitor was included as a control. If the propofol-induced developmental toxicity and apoptosis were rescued after zebrafish embryos were treated with inhibitors of pro-apoptotic genes in the endogenous pathway, the exact mechanism could be established, which will be investigated in our future work. Nevertheless, this model allowed us to carefully investigate the apoptotic effect of developmental toxicity induced by propofol and further develop possible protective strategies.

## Conclusion

Our study found that zebrafish embryos exposed to propofol exhibited significant growth retardation and organ damage, which might be related to propofol-induced expression of *casp3a*, *casp3b*, *casp9*, and *baxb* in the endogenous apoptotic pathway. However, further research on the effects and mechanisms of anesthetic drugs on development is required, focusing on specific apoptotic molecular target inhibitors. Our determination of the mechanism of propofol-induced apoptosis in developing zebrafish is expected to provide a valuable basis to explore the effects of anesthetics on children in non-obstetric and neonatal surgery, and improve the related clinical safety of anesthetics.

## Supporting information

S1 Fig Morphology of zebrafish and deformities induced by propofol in each period. (A-C) The 1 day post fertilization (dpf) zebrafish. (D-E, G-H) The 2 dpf zebrafish. (F, I-N) The 3 dpf zebrafish. (O-P) The 4 dpf zebrafish. (B-D, G, I-J, O) The zebrafish in the control group. (A, E-F, H, K-N, P) All the zebrafish in the 2 μg/ml propofol group showed less pigmentation and shorter body length than the normal zebrafish at the same developmental stage. (A) The dead embryos were organized into a flocculent mass. (B) The caudal fin did not develop. (F) The non-hatching 3 dpf embryos in the 2 μg/ml propofol group showed serious malformation of heart development and edema. (H) The 2 dpf embryos exposed to propofol were manually decapsulated under the microscope to show yolk sac hemorrhage. (K) Yolk sac hemorrhage and edema occurred in the 3dpf larva after propofol exposure. (M-N) 3dpf larvae displaying abnormal spine curvature from a back and lateral view. Black arrows represent yolk sac hemorrhage and hollow arrows represent edema. Scale bar = 1 mm. Magnification = ×63 (A-F), ×32 (G–N), ×25 (O-P).
(TIF)

S2 Fig The details of terminal deoxynulceotidyl transferase nick-end-labeling (TUNEL) staining. Fluorescein isothiocyanate (FITC) (nucleus), 4′,6-diamidino-2-phenylindole (DAPI) (apoptotic cells) and merged images are shown. The white scale bar (1 mm) belongs to the 72 hours post fertilization (hpf) larvae; the black scale bar (1 mm) belongs to the others. Magnification = ×40.
(TIF)

S3 Fig Apoptotic gene expression without significant changes induced by propofol. (A) Pro-apoptotic gene expression. (B) Anti-apoptotic gene expression. An independent sample t-test was used to compare the mRNA expression in the two groups at the same time point. Three independent experiments were carried out. *p < 0.05, **p < 0.01, ***p < 0.001, ****p < 0.0001.
(TIF)

S1 Table Sequences of primers used for quantitative real-time reverse transcription-PCR. (DOCX)

S2 Table Sequences of primers used for whole-mount in situ hybridization. (DOCX)
