# Supplementary figures and images for: Apoptotic mechanism of propofol-induced developmental toxicity in zebrafish embryos
Source: PLoS One. 2023 May 30;18(5):e0286391. doi: 10.1371/journal.pone.0286391 (PMC10228783; doi:10.1371/journal.pone.0286391)

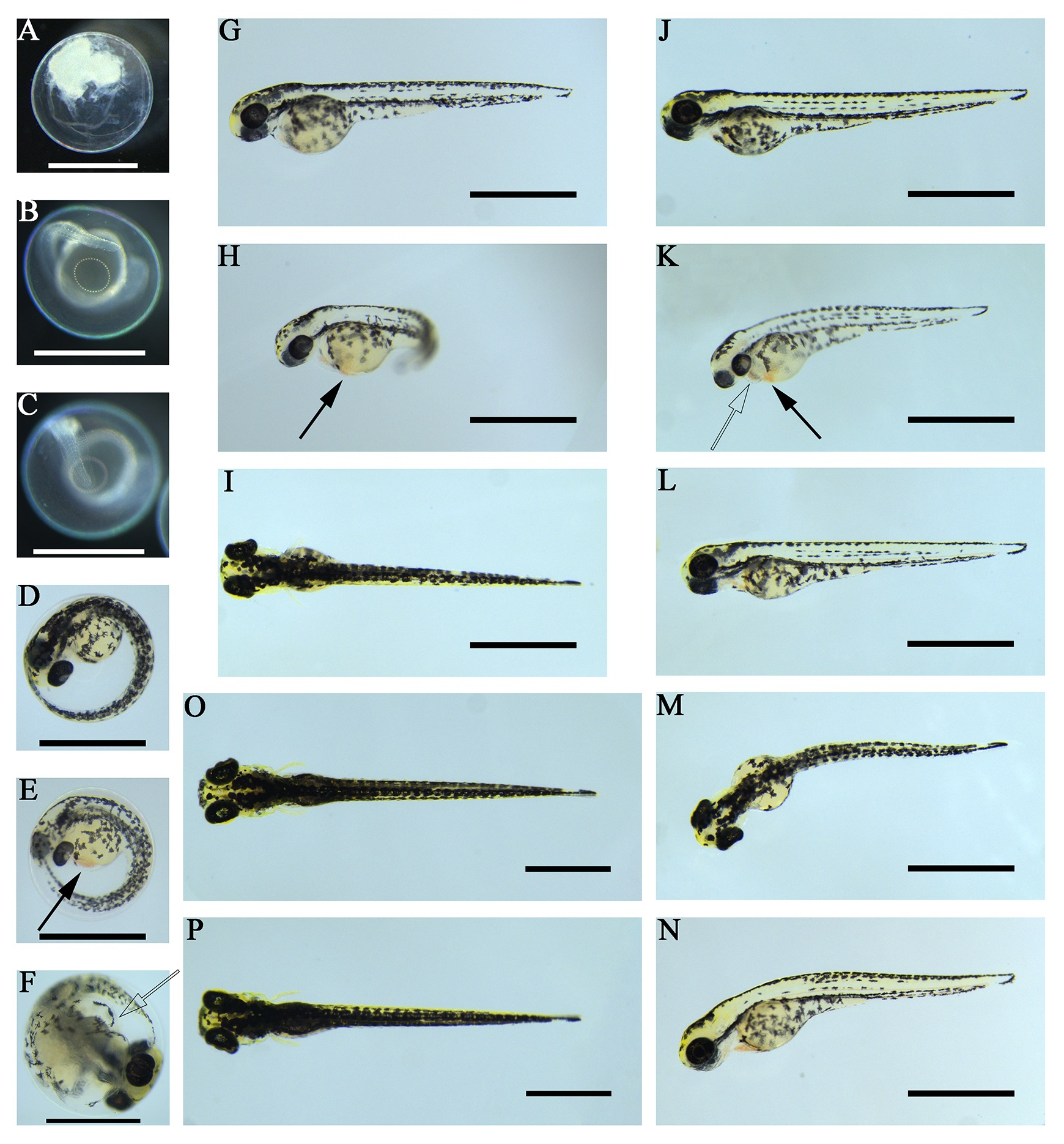

Supplement: S1 Fig — (A-C) The 1 day post fertilization (dpf) zebrafish. (D-E, G-H) The 2 dpf zebrafish. (F, I-N) The 3 dpf zebrafish. (O-P) The 4 dpf zebrafish. (B-D, G, I-J, O) The zebrafish in the control group. (A, E-F, H, K-N, P) All the zebrafish in the 2 μg/ml propofol group showed less pigmentation and shorter body length than the normal zebrafish at the same developmental stage. (A) The dead embryos were organized into a flocculent mass. (B) The caudal fin did not develop. (F) The non-hatching 3 dpf embryos in the 2 μg/ml propofol group showed serious malformation of heart development and edema. (H) The 2 dpf embryos exposed to propofol were manually decapsulated under the microscope to show yolk sac hemorrhage. (K) Yolk sac hemorrhage and edema occurred in the 3dpf larva after propofol exposure. (M-N) 3dpf larvae displaying abnormal spine curvature from a back and lateral view. Black arrows represent yolk sac hemorrhage and hollow arrows represent edema. Scale bar = 1 mm. Magnification = ×63 (A-F), ×32 (G–N), ×25 (O-P). (TIF) [file pone.0286391.s001.tif]

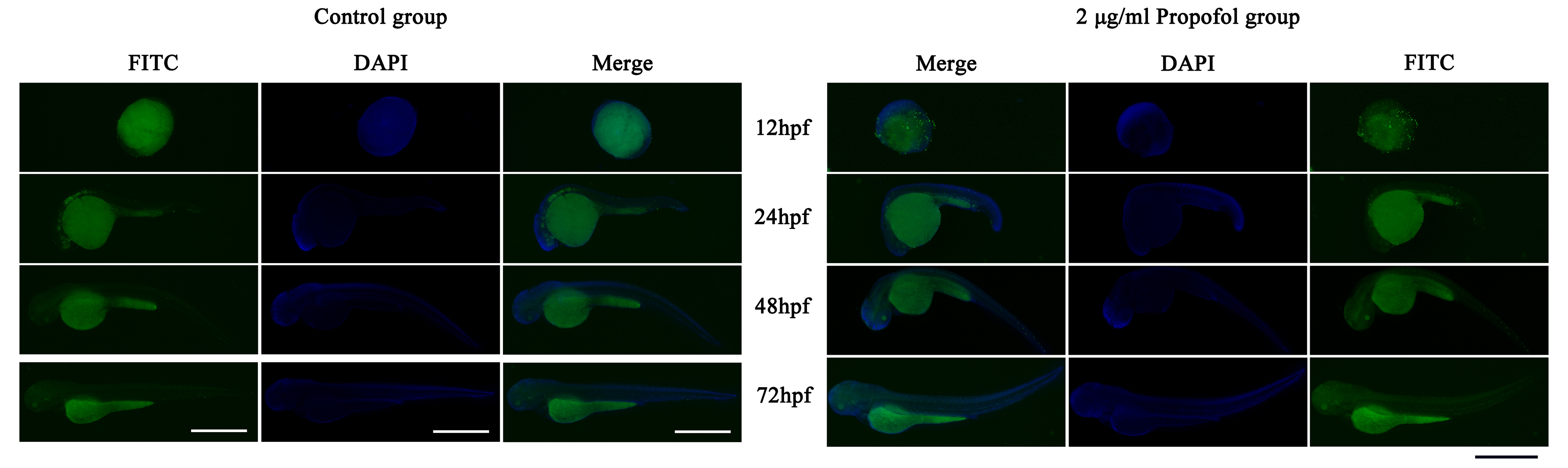

Supplement: S2 Fig — Fluorescein isothiocyanate (FITC) (nucleus), 4′,6-diamidino-2-phenylindole (DAPI) (apoptotic cells) and merged images are shown. The white scale bar (1 mm) belongs to the 72 hours post fertilization (hpf) larvae; the black scale bar (1 mm) belongs to the others. Magnification = ×40. (TIF) [file pone.0286391.s002.tif]

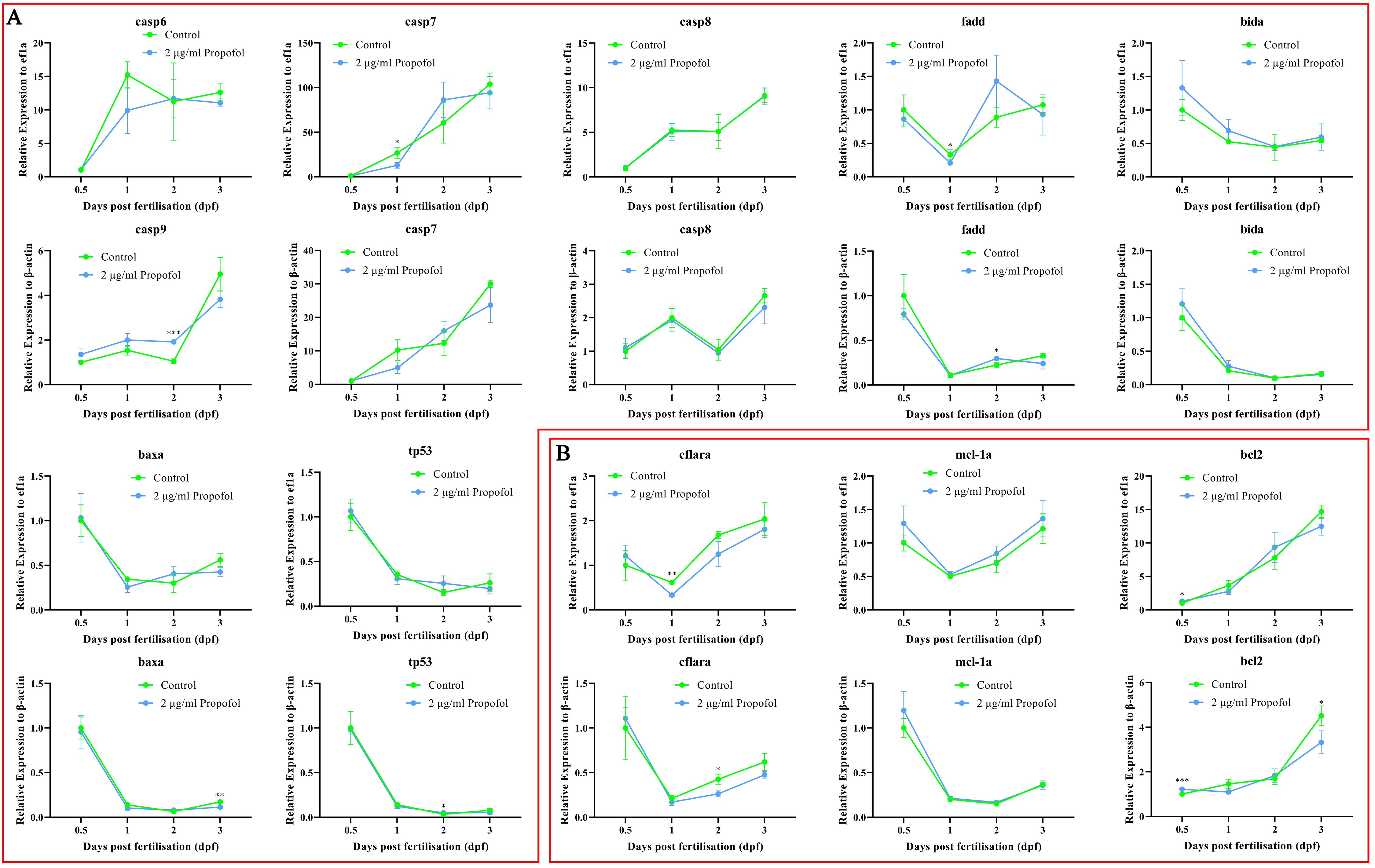

Supplement: S3 Fig — (A) Pro-apoptotic gene expression. (B) Anti-apoptotic gene expression. An independent sample t-test was used to compare the mRNA expression in the two groups at the same time point. Three independent experiments were carried out. *p < 0.05, **p < 0.01, ***p < 0.001, ****p < 0.0001. (TIF) [file pone.0286391.s003.tif]
