# Supplementary material for: Apoptotic mechanism of propofol-induced developmental toxicity in zebrafish embryos
Source: PLoS One. 2023 May 30;18(5):e0286391. doi: 10.1371/journal.pone.0286391 (PMC10228783; doi:10.1371/journal.pone.0286391)
Supplement: S1 Table — (DOCX) [file pone.0286391.s004.docx]

| Gene Name | Forward primer 5′- 3′ | Reverse primer 5′- 3′ | NCBI ID |
| --- | --- | --- | --- |
| casp3a | AAGCGTGTGGATACAACAGATGCTA | CCATTGCGTGGGTTCATGC | NM_131877.3 |
| casp3b | TGGACTCGTTCAGATGGATGC | CGAACCCCCATTCCTGTTCT | NM_001048066.2 |
| casp6 | CAGCAGGACAGACAGTTGAAGA | GAACGGTAGCCCAATCCCAG | NM_001020497.1 |
| casp7 | CCTGACCGCAAAGGGAGATT | ACGTACATTCATCCCTGTCTTT | NM_001020607.1 |
| casp8 | CAGAGACCAGGAACAAGGAGG | TAATTGTGCCAGCCGAAGAGT | NM_131510.2 |
| casp9 | ACAAGACATATAGTCCACCTTCTGA | GCTTGTCGCAGTCGATGTTG | NM_001007404.2 |
| tp53 | GAACCCCGGATGGAGATAACTT | CACAGTTGTCCATTCAGCACC | NM_001328588.1 |
| baxa | ACTTTGCCTGTCGCCTTGTC | TAACTGCGGATTCCGTCCCATC | NM_131562.2 |
| baxb | CACAGTAGGAGGATGGATGAACA | ACTTGTGTGCCATCTCCATTC | NM_001013296.2 |
| bida | TCAGCCTGGTCTTTCAGTCAA | AGATGACTGGCCCAAACCTG | NM_001079826.1 |
| bcl2 | GGGGCGGATCATTGCATTCT | CACGAAGGCATCCCAACCTC | NM_001030253.2 |
| bcl2l1 | CAAGGAGGATGGGAACGCTT | TTCTGTGCAATGAGTCCCCC | NM_131807.1 |
| mcl-1a | GCGATACTCGGCAGCTCTTA | GAAGACGCTGGATCATTCCTTT | NM_131599.1 |
| mcl-1b | CATGGACACGCGGGAGATTA | AGCCGTGCAATCATACCTTTG | NM_194394.2 |
| fadd | CGCTGATCTCGTGGAGAGAAA | AAAAGCTGTGCTCGACTCCT | NM_001386360.1 |
| pmaip1 | CGAAGAAAGAGCAAACCGCT | AGCAGGTACAGATCCCTTCTCA | NM_001045474.3 |
| bbc3 | TTCCTTCAGAGGAATGCCGT | GCACTCGTCCTGTCCTTTGT | NM_001045472.2 |
| cflara | TGAAAGGACATGAGAGAAATGTGC | ATGGAGTGGTTTGTGTTGTGTTG | NM_001313772.1 |
| actb1 | CTCTTCCAGCCTTCCTTCCT | CTTCTGCATACGGTCAGCAA | NM_131031.2 |
| ef1a | GATGCACCACGAGTCTCTGA | TGATGACCTGAGCGTTGAAG | NM_131263.1 |
