# Supplementary material for: Apoptotic mechanism of propofol-induced developmental toxicity in zebrafish embryos
Source: PLoS One. 2023 May 30;18(5):e0286391. doi: 10.1371/journal.pone.0286391 (PMC10228783; doi:10.1371/journal.pone.0286391)
Supplement: S2 Table — (DOCX) [file pone.0286391.s005.docx]

| Gene Name | Direction | Primer 5′- 3′ | NCBI ID |
| --- | --- | --- | --- |
| casp3a | Forward | GATCGCAGGACAGGCATGAA | NM_131877.3 |
|  | Reverse | TAATACGACTCACTATAGGGGAACAGAGGCAAGTGAAACCG |  |
| casp3b | Forward | TCATCTCACACACAGATTGACCA | NM_001048066.2 |
|  | Reverse | TAATACGACTCACTATAGGGTCTCGCAACTTAATCAGTTAAATCC |  |
| casp9 | Forward | TCAGCGCAGGCATGATGTAA | NM_001007404.2 |
|  | Reverse | TAATACGACTCACTATAGGGTCGATTATGGCTTGCCTCAGT |  |
| baxb | Forward | AGGGGTAGTAAGGCAGGAGC | NM_001013296.2 |
|  | Reverse | TAATACGACTCACTATAGGGCAAGAGAGTCGACGAAACAGGT |  |
